# The bumpy ride to a medical PhD degree: a qualitative study on factors influencing motivation

**DOI:** 10.1186/s12909-023-04973-z

**Published:** 2024-02-19

**Authors:** C. R. den Bakker, B. W. C. Ommering, A. J. de Beaufort, F. W. Dekker, J. Bustraan

**Affiliations:** 1 Center for Innovation in Medical Education, Leiden University Medical Center, Hippocratespad 23, Zone V7-P, PO Box 9600, 2333 ZD Leiden, The Netherlands; 2 Research Centre for Learning and Innovation, Research Group On Research Competence, HU University of Applied Sciences Utrecht, Utrecht, the Netherlands; 3 Department of Clinical Epidemiology, Leiden University Medical Center, Leiden, the Netherlands

**Keywords:** Medical PhD programmes, Doctoral education, Motivation, Self-determination theory, Clinician-scientist, MD-PhD

## Abstract

**Introduction:**

In parallel with a tremendous increase in medical PhD enrolments, concerns have risen about PhD candidates’ poor well-being, increasing attrition rates for PhD programmes, and, eventually, a decline in clinician-scientists. According to the Self-Determination Theory, autonomous motivation is strongly linked to positive aspects of well-being and other positive outcomes such as study completion and success. In this way, motivation has a pivotal role in successful completion of medical doctoral programmes. In this study we explored factors affecting motivation during the PhD journey and aimed to contribute to engaging doctoral education environments, and, eventually, a sustainable clinician-scientist workforce.

**Methods:**

This constructivist qualitative interview study was conducted among ten medical PhD candidates in the final phase of their PhD. We used timeline assisted interviews to identify meaningful experiences throughout their PhD journey. Thematic analyses as an iterative process resulted in overarching themes.

**Results:**

We identified six themes influencing autonomous and controlled motivation along the challenging PhD journey: (1) Initial motivation to start a PhD matters; (2) Autonomy as a matter of the right dose at the right time; (3) PhD as proof of competence and/or learning trajectory?; (4) It takes two to tango; (5) Peers can make or break your PhD; (6) Strategies to stay or get back on track.

**Conclusion:**

This study revealed factors that contribute positively and/or negatively to autonomous and controlled motivation. Some factors impacted motivation differently depending on the PhD phase and individual strategies. Additionally, some factors could coincide and change from positive to negative and vice versa, showing that a successful journey cannot simply be reduced to an absence of negative experiences.

**Supplementary Information:**

The online version contains supplementary material available at 10.1186/s12909-023-04973-z.

## Introduction

Medical PhD programmes aim to train future generations of clinician-scientists i.e., medical doctors who combine patient care with research. Enrolment in medical PhD programmes has increased tremendously in the past decades [1–5]. Simultaneously, there are concerns about PhD candidates’ well-being [6–10], a complex combination of the presence of positive (e.g. satisfaction, self-efficacy, work engagement) and/or absence of negative (e.g. anxiety, stress, burnout) mental states [11]. Several studies found that 30–50% of PhD candidates self-report significant levels of stress, burnout and other mental health problems [12–16]. Negative aspects are related to delaying doctoral study and intentions to quit [17–23]. Subsequently, programme attrition, with rates between 25–60%, is a major concern in the medical doctoral domain, as well as in other doctoral domains [10, 19, 24]. This issue is particularly critical as it may potentially contribute to the decline in and shortage of clinician-scientists [25, 26].

Motivation is strongly linked to well-being and, hence, persistence and study completion and success [6, 27–31]. Therefore, insight into factors affecting motivation of medical doctors (MDs) pursuing a PhD could provide guidance on how to optimize medical doctoral programmes’ learning environments and supports in maintaining and fostering motivation during the programme. In this study, motivation is regarded as a multidimensional construct consisting of different types of motivation based on Self-Determination Theory (SDT) [27–30]. SDT distinguishes autonomous and controlled motivation. *Autonomous motivation* (AM) derives from a PhD candidate attributing personal value to learning, due to genuine interest and pleasure in the research itself. *Controlled motivation* (CM) includes persuasion of learning or work as a means to an end that is separate from the activity itself, for example to obtain a reward such as a future training or job position. Autonomous motivation is associated with positive outcomes in education, such as intention to persist and subjective well-being, whereas controlled motivation is reported to be associated with negative outcomes, such as anxiety and lower positive affect [6, 28, 31–33].

A PhD in the medical field is more common than in any other domain [19]. Furthermore, the research environment of medical PhDs differs substantially from environments in other fields. Medical PhD candidates are (future) medical doctors, who commonly combine patient care with their PhD trajectory, mainly supervised by PhD-holding clinicians, and often return to clinical care after their PhD trajectory [34]. Furthermore, as they are employed at a clinical department, the healthcare culture and hierarchy will affect the research environment. In addition, some programme directors consider a PhD highly important or necessary to get a specialty training position [35]. To this end, a subset of MDs obtains a PhD degree to gain admission to their desired specialty [36]. This admission-related aspect of pursuing a PhD might be more prevalent in medicine in contrast to domains and, by definition, is controlled motivation.

Recently, we quantitively explored autonomous and controlled motivation and its relation to work engagement, (expected) delay, drop-out intentions, and clinician-scientist career ambitions in over 1300 Dutch medical PhD candidates^1^. Our national survey study showed that autonomous motivation was positively related to PhD candidates’ work engagement and clinician-scientists career ambitions. In addition, higher autonomous motivation resulted in less drop-out intentions, contrary to controlled motivation which was related to lower work engagement and research ambitions, and higher drop-out intentions. However, insight into factors affecting autonomous and controlled motivation during the PhD journey was lacking and deeper understanding called for a qualitative approach. In this follow-up study we aim to answer the question of which factors affect autonomous and controlled motivation during the PhD journey. By that, we aim to contribute to the conscious use of strategies to increase autonomous motivation and, hence, well-being, successful completion of the PhD programme, and, eventually, a sustainable clinician-scientist workforce.

## Methods

### Study design

For our interview study, we used a constructivist approach. A constructivist paradigm asserts that knowledge and reality are socially constructed by people through experiences and reflections on those experiences, and that researchers should attempt to relate to subjective experiences of study participants [37]. Interviews are a commonly used method within the constructivist paradigm and, in our view, match well with our aim to understand how, when and why PhD candidates’ motivation develops during their PhD trajectory. We designed a guide (Additional file 1) for semi-structured, timeline-assisted interviews that were held between April and July 2021. Timelining adds a chronological visual representation related to the experience, anchors the interview and helps the participant to identify and focus on meaningful events and experiences. It can provide participants a way to reflect deeply on their stories and even help to create new understandings [38, 39]. Interviews started with open questions about the interviewee’s pathway prior to their start as PhD candidate. When participants reached the start of their PhD trajectory in their story they were asked to write meaningful experiences of their PhD trajectory (e.g. persons or events) down on post-its. Hereafter, they were asked to put these experiences on their PhD trajectory timeline as tool for reflection. To gain more insights into the impact of these experiences on their motivation during their PhD, participants were asked to position post-its that had greater positive impact on their motivation higher on the y-axis. During the rest of the interview, experiences were chronologically discussed in-depth and the PhD timeline was reflected on.

### Study setting

In the Netherlands, there are different pathways to embark on a PhD trajectory. After graduation and before applying for a specialty training position, junior doctors mostly choose to either work as a doctor-not-in-training to gain more work experience, or to apply for a PhD position before or after gaining clinical work experience. Less common pathways are obtaining a PhD as medical student (MD-PhD track), as resident already in training, or later as medical specialist. PhDs are (mostly paid) employees facilitated at a University Medical Center (UMC)^1,^^2^.

### Sampling and data collection

PhD candidates with a master’s degree in medicine and in the final phase of their PhD trajectory at various departments of all Dutch medical graduate schools were selected using purposive sampling to include a variety of participants with different motivational profiles. Selection was based on relatively low and high AM and CM scores (based on population mean; three PhD candidates with relatively low AM and high CM, four PhD candidates with relatively high AM and low CM, three PhD candidates with relatively high AM and high CM) and different pathways (i.e. five participants not in training with clinical working experience as doctor not in training, two participants not in training without clinical working experience as doctor not in training, one participant combining working as doctor not in training with a (unemployed) PhD trajectory, and two participants who were residents in training) as found in our previous national survey study^1^. Participants were affiliated with diverse medical specialties (internal medicine, plastic surgery, ENT (Ear, Nose, and Throat), orthopaedics, gynaecology and obstetrics, and surgery), and various Graduate Schools, connected to all Dutch university medical centres. Participants were invited by email and all agreed to participate. The first author (CdB) conducted ten interviews of 60–90 min until inductive thematic saturation (i.e. the point when additional data leads to no new emergent codes or themes) was achieved [40]. All interviewees verbally consented participation and audio-recording before the interview started. They were informed that pseudonymized data would only be accessible for co-authors and that published results would be strictly anonymous. Sampling and data collection occurred concurrently with thematic analysis and informed future data collection.

### Data analysis

Interviews were audiotaped, transcribed verbatim and pseudonymized. The interviews were analysed using thematic analysis [41]. Two researchers (CdB and JB) independently conducted open coding using Atlas.ti. Similar codes were grouped under coding categories and then moved from the categorical level (open codes and categories) to the conceptual level (relationships between codes and construction of important themes), an iterative process using an inductive approach [42]. Through ongoing discussions, consensus on the coding scheme was reached. There were several meetings (CdB, JB, BO) to discuss overarching themes and to ensure that the research question was addressed adequately. Methodologic rigor was strengthened through triangulation in data analysis (i.e. independent data analysis by two investigators followed by team discussions and consensus) and member checking to ensure that interpretations were accurate [43]. 

### Research team & reflexivity

Our multidisciplinary research team included members with a variety of backgrounds and perspectives. CdB is an MD and PhD candidate in medical education. For interviewees she was considered as a peer without conflict of interest as she was not employed within a medical specialty, which helped to create a safe environment to talk openly about PhD experiences. JB has a background in educational sciences, and is a senior consultant and researcher in postgraduate medical education. The other authors are experienced educational researchers and PhD supervisors with backgrounds in pedagogical and educational sciences (BO), paediatrics (AJdB), and clinical epidemiology (FD). The diversity of backgrounds and expertise within the team enhances the trustworthiness of our results. All researchers were familiar with SDT prior to this study as it was used as framework for our earlier studies. In line with the constructivist approach to reality, we were well aware of the role of this theory including the general concepts of AM and CM. Yet, to take into account the in-depth, exploratory character of this interview study, we explicitly chose not to deliberately start looking how relatedness, autonomy and competence played a role in the development of our interviewees’ motivation, which is why we choose a timeline approach where participants were free to share what came to mind. In this way, we consciously aimed to be as open as possible to all themes coming up during the interviews or (open) coding process. 

We used the COREQ-32 checklist to report important aspects of the research team, study methods, context of the study, findings, analysis and interpretations [44].

## Results

Motivation throughout a PhD journey developed simultaneously with meaningful events. Our analysis revealed six themes. Within these themes, sub-themes provide further insight into factors affecting autonomous and controlled motivation (AM and CM) during a PhD trajectory. Because of the rich data, not all subthemes are discussed in detail. An overview of all themes and subthemes can be seen in Additional file 2. The following higher-order themes emerged:

1. *Initial motivation to start a PhD matters*. Motives to start a PhD are already formed prior to enrolment influenced motivational development throughout the PhD journey. Most candidates stated that the option to start a PhD trajectory happened to ‘come their way’, e.g. while working as doctor-not-in-training, without actively looking for a PhD programme. We identified three main reasons to embark on a PhD trajectory, which can be categorised from high to low AM:
  1.1 *As stepping stone towards a clinician-scientist career*. A PhD trajectory was started with a genuine interest in research. Participants described the desire to (1) immerse themselves into a topic that they were passionate about, (2) become an expert on a specific topic, and/or (3) have an opportunity to be challenged in critical and creative thinking as this was perceived by some as insufficient in their clinical job, with many protocols and standardized procedures.
    ‘You learn little about research in medical school. It is just an education that really makes you primarily become a doctor, but not so much a scientist. So I really wanted to learn that. Actually getting a kind of driver's license for doing scientific research, that's how you might put it.’ – Interviewee #7
  1.2 As stopover for career orientation purposes. This motivation often was stated with a short term future perspective. Research was perceived as (potentially) interesting and fun, but a PhD trajectory was used to buy time for considering future career steps, mature further, have a break from the clinics and/or as career orientation for the long term in both the clinical and scientific world.
    ‘Firstly, because it seemed good just for my CV and by that, I also thought it would be a better way to obtain a specialty training position. Furthermore, I also wanted to give myself some time to do something totally different.’ – Interviewee #9.
  1.3 *As vehicle to gain admission to future clinical job positions*. A PhD trajectory was used to improve chances for admission to the preferred specialty. It was considered useful for network contact and perceived as a prerequisite to get a training position within the specialty. Genuine interest in research and/or the research topic were less relevant.
    ‘And a lot of people also strategically opt for a PhD programme in which as little effort as possible is needed and which is completed as soon as possible.’ – Interviewee #5
  In most cases, multiple reasons coexisted. Additionally, motives to start a PhD were often supplemented with the ‘why not?’ argument, in which a PhD trajectory was valued as something that can only benefit and won’t harm you. While motivation can change over time, the motives for initiating a PhD were indicative and mattered for coping strategies during meaningful events throughout the PhD, especially in the first phase.
2. *Autonomy, a matter of the right dose at the right time*. Candidates perceived autonomy in research activities as a need during the programme. This need appeared to vary throughout different phases during the PhD trajectory. PhD candidates stated that, in the first phase, they often felt consciously incompetent, resulting in a stronger need for guidance than autonomy, whereas at a later stage the need for autonomy became enhanced. If the ‘autonomy dose ‘ needed at a certain stage was insufficiently met, frustration ensued and negatively impacted AM.
    ‘I think it's very important that people know where to go to when having questions. Not like you’re swimming in the deep, forever, because no one tells you what the plan is. You really don’t know anything at the beginning of your PhD.’ – Interviewee #6
    ‘So when it (i.e. the research projects) started to take off and I got more and more of an idea what my PhD entailed and where it should go, my motivation also went up sharply.’ – Interviewee #8
  In contrast, the importance of autonomy in working hours and not working shifts did not vary throughout the PhD trajectory and resulted in improved work-life balance and enhanced motivation.
3. *PhD as proof of competence and/or as learning trajectory?* Most PhD candidates considered their PhD period as a learning trajectory. However, some believed that supervisors perceived the trajectory as proof of competence. PhD candidates then assumed that they were expected to already master and show sufficient skills to succeed in the research tasks assigned to them right from the start.
    ‘There is a lot of competition around you, so you also have to work very hard to keep up with that and show that you are worth it and you can surely show that within a PhD programme, because you can show that you are able to achieve things.’ – Interviewee #6
  This ‘fear of failure’ was fostered in a dependency relationship and mainly resulted in imposter syndrome; an internal experience of believing that you are not as competent as others (i.e. supervisors) perceive you to be and not willing to fail in the eyes of the supervisor. This leads to feelings of self-doubt, feeling lost, and loneliness.
    ‘And she (i.e. supervisor) literally thought that I should be able to do it all on my own and I disagreed and that made it difficult.’ – Interviewee #1
  These feelings often led to a decrease in both AM and CM and could result from and/or be further strengthened by expected supervisor’s beliefs. Vice versa, supervisors were able to foster confidence and self-efficacy and, accordingly, counteract the imposter syndrome.
4. *It takes (at least) two to tango*. Supervision is a process that aims to support and assure the development of knowledge, skills and values of PhD candidates. According to PhD candidates, this requires a supervisor who is approachable, makes time, provides constructive and timely feedback, gives trust and autonomy, and sees the person behind the research projects.
    ‘We also have conversations, more on a kind of meta level about the professional development of a young doctor or clinician-scientist. That goes beyond just discussing research content. That's great, because it just works well and is very good and important, I think, for a successful and pleasant PhD trajectory.’ – Interviewee #7
    ‘My co-supervisor was really - that's what I'm trying to emulate now – on how to guide someone - and we also guide students together. Just very positive, always available to spar with, always responding to me within a week with good suggestions and good feedback. And just encouraging, so giving positive feedback, says “well done”, always being positive in emails, and so on, so he's really a great supervisor.’ – Interviewee #8
  Supervision can be provided by the thesis-promotor and/or by other research team members. PhD candidates perceived supervision as one of the most crucial factors for their motivation. A good fit with at least one supervisor was key to their AM as it directly affected their autonomy and self-efficacy, and vice versa; a lack of a good fit resulted in negative feelings as stress, loneliness, incompetence and frustration.
    ‘There was little input or guidance from them. I expected a bit more involvement in the process I'm going through or the research I'm doing, but it was quite disappointing. I quickly got the feeling of, do you really care about the work I'm doing? But well, maybe that's not what they wanted to convey, but that's the feeling I got anyway. – Interviewee #3
    ‘So there was more pressure on me to publish and show results, and I actually had to do it all on my own without any guidance. So that wasn't communicated well by the supervisors, that I had to do it all on my own and that I actually had to be able to do it all before starting the PhD. (…) In retrospect, I think that the supervisor and I just didn't fit each other and that it didn't work from the beginning.’- Interviewee #1
  An additional good fit with other supervisors was beneficial, but not as crucial as a good fit with at least one supervisor ‘to tango with’.
5. *Peers can make or break your PhD*. Peer support was important on different levels for enhancing AM. Peers, mostly PhD candidates from the same department or research group, could share their experiences. Professionally, this was useful in sharing resources and effective strategies. On a personal level, peers countered feelings of loneliness or social isolation and provided support in personal doubts, e.g. career orientation. Peer activities in non-formal settings, for example during an international conference trip or Friday drinks, facilitated peer support.
    ‘The most important thing about a PhD trajectory is that you get a really special bond with your peers who you work with day by day. (...) because of your colleagues, I think you are able to hold on, they are a great support. They make it (i.e. PhD trajectory) the most fun.’ – Interviewee #1
  The lack of peer support, e.g. within a competitive context or due to drop-out of peers, resulted in an unsafe learning environment and negatively impacted AM.
    ‘In any case, negative things are rarely discussed because you do not want to give the impression that you- that things are not going well or…- status is just so important. You just have to be in control and you have to do things with great pleasure.’ – Interviewee #3
6. Strategies to stay or get back on track. PhD candidates experienced the trajectory as a bumpy and challenging ride with highs and lows. These ‘bumps’ were often assumed to be part of the PhD journey, for example slow progress, dealing with ‘politics’ (e.g. conflicting interests with supervisors, or authorship issues), disappointing research outcomes, and no good fit with the research team. In case of frustration in needs, or conflicting values or interests, PhD candidates used two types of approaches to keep going and stay or get back on track:
  6.1 *Active solution-seeking approach*. PhD candidates actively sought workarounds to overcome struggles and keep going. They used solution-seeking strategies such as ‘speaking up’ and ‘making some changes’, for example by continuing their work at another work place or department, by finding peers for personal support, or actively seeking for collaborations or supervision elsewhere, to change the team into ‘a winning team’. When PhD candidates successfully conquered the ‘bumps’, feelings of achievement, personal growth, and eventually, AM was fostered.
    ‘I just really missed having a sparring partner and I couldn’t get that from her, so I had to look for it somewhere else. (...) And I am now glad that I got through this low point and got closer to my own values and norms.’ – Interviewee #1
  Most PhD candidates who aspired to a future research role explicitly mentioned they definitely wanted to use and translate their own learning experiences (varying from good to bad) in how they would fill in their future role as research supervisor. Lastly, dependency was considered a risk factor for conflicts with personal values to avoid professional conflicts. An often mentioned barrier to protect personal values and/or speak up was the vulnerable position in which most PhD candidates are in, for instance when they admire to obtain a desired job position while supervisor(s) or other colleagues had powerful roles (e.g. programme director) in this procedure.
    ‘Everyone was totally – people had become a bit cynical due to the work and workload, feeling unheard and being in a dependency position for obtaining a desired specialty training position, so they couldn’t speak up. And that was such a big adjustment that in the beginning, I really thought “Oh, what have I gotten myself into?”. But you just keep going and eventually you get used to it. I also started sitting somewhere else, with another group with a more positive vibe.’ – Interviewee #3
  6.2* Accept that lows are part of a PhD journey*. PhD candidates accepted that lows were part of their PhD and used (passive) ‘take it or leave it’ coping strategies to stay motivated. This ‘tendency to accept’ was stronger when PhD candidates were dependent on their supervisor(s) to get a desired future career position. This was a sustainable strategy when, for example, a highly desired specialty training position was obtained; it was all worth it in the end.
    ‘I was able to accept pretty soon that those are external factors that you just have to resign yourself to, because you simply can't do anything about it.’ – Interviewee #9
    ‘But I took that for granted, because I also thought, well; I just have to persevere, as soon as I’m a resident things will get better again. So you go on and you accept it. (…) But yes, I have invested so many hours that I just really want to finish it now.’ – Interviewee #10
  However, when the ‘wheels fell off’ and the desired job position was not obtained or no longer wished to obtain, frustration replaced genuine interest and joy and mainly CM was a source to keep going. In addition, PhD candidates also used this strategy as they did not want to give up because they have come this far and already invested a lot of time and energy (‘sunk cost effect’;* i.e. the tendency to persist in a decision, even when it is unfavourable, because it involved significant costs as time, money and/or effort*) and/or they do not want to disappoint themselves and others.
    ‘I had never realized before, but in research it all has to do with who has the most power? Who is in charge? There will be authors on papers who have actually done nothing, but purely as favour. You have to work with people just to satisfy people and it's usually not the best for the research, we don't get the best results from that. But unfortunately that's how it goes...’ – Interviewee #5
    ‘Once I start something, I want to finish it. And that feeling was much stronger than, well, you know; I don't want to get into that desired specialty anymore, so I'm not going to get my PhD anymore either.’– Interviewee #1
  When candidates mainly mentioned negative experiences (e.g. conflicts with personal values) when reflecting on their timeline, while at the same time over years a great effort was spent to achieve the PhD degree, they often added that, in hindsight, it was worth the effort. They described it to be valuable for other important aspects such as personal development, friendships that emerged, or career progress and orientation (in both specialty and academia).
    ‘Well, it obviously moulds you into the person you are now. It's hard to then…That six months abroad gave me so much, also on a personal level, so many insights and that was such a cool period that – even though it was a hard time afterwards – it was worth it.’ – Interviewee #3
    ‘But it (i.e. PhD trajectory)– even though I may sound a little negative overall – has also brought me good things. So I did really enjoy doing it as well. (…) Well, maybe I want to emphasize that I don't want to say…It hasn't been a very negative experience, but it's how I look back on it now and it hasn't been like that over all these years.’ – Interviewee #10

## Discussion

Insights into factors affecting PhD candidates’ motivation during their PhD journey are useful for both PhD candidates and their supervisors. The theoretical concepts of autonomous motivation (AM) and controlled motivation (CM) underpin the motivational dynamics. AM reflects internal motivation driven by personal interest and satisfaction, while CM arises from external factors. We identified six themes influencing AM and CM along the challenging PhD journey: motives to start a PhD, autonomy at the right dose and time, a PhD trajectory to be a proof of competence and/or learning trajectory, support from supervisors and peers, and strategies to stay or get back on track.

Most studies on PhD candidates’ experiences focused on negative attributes such as stress, anxiety, depression, and burnout, while positive aspects of a PhD experience have been studied to a lesser extent [6, 8–11, 16, 23, 45]. This study reveals that positive and negative motivational factors of PhDs coincide as some factors were experienced positively, while the opposite was being experienced negatively, and vice versa (e.g. a good supervisor and the lack of a good supervisor). Some factors impacted motivation differently over time, changing from positive to negative and vice versa (e.g. dose of autonomy). In addition, there are individual differences in how a factor is perceived, showing that a successful journey cannot be simply reduced to just an absence of negative experiences. A recent single-center study on both energizers and stressors of medical PhDs provided a first insight into factors affecting a PhD journey in medicine [34]. Our national multi-center interview study adds, in addition to in-depth insight into factors affecting motivation during a PhD, that factors such as the dose of autonomy can contrary affect motivation depending on both the phase of the PhD and, in the end, individual strategies. Hence, one size fits nobody when it comes to supporting and maintaining an individual PhD’s motivation. This underlines the relevance of reflecting on these themes before and during the PhD programme and to adjust support based on the outcomes of this reflection. Making the implicit explicit could contribute to AM and hence, well-being, successful PhD completion, and, eventually aspired (future) clinician-scientists.

PhD candidates are usually high achievers, especially in the medical field when next to a research pathway a clinical career is aspired to [46]. Coping strategies such as ‘finish what you start’ or ‘keep your eyes on the prize’ were mentioned frequently and linked to an increase in CM. In addition, the concept of cognitive dissonance might be at stake in cases where some PhD candidates clearly described downsides of their PhD trajectory, yet had a tendency to quickly narrow down these as well. Cognitive dissonance refers to a situation involving conflicting attitudes, beliefs or behaviours resulting in feelings of discomfort leading to an alteration in one of the attitudes, beliefs or behaviours to reduce the discomfort and restore balance. Furthermore, distressing feelings arise when a PhD is perceived as a proof of competence and contributed to CM. Particularly in the first phase of the PhD, when self-efficacy levels are often low, these feelings are linked to the imposter syndrome. Stelling et al. found that the imposter syndrome among early career clinicians is associated with burnout as a result of ‘striving to fit in and stand out’ [47]. Sverdlik et al. studied the imposter syndrome among doctoral students and found that feelings of belonging were a negative predictor of imposter syndrome which, in turn, predicted higher levels of depression, stress, and illness symptoms [45]. In line with these studies, our study highlights the importance of fostering a supportive environment. To foster AM, our results show that this support is important at different levels (i.e. academic, autonomous, and personal level), which is also described by Overall et al. [48]. Support on the academic and autonomous level is mainly fulfilled by the research team and highly dependent on feeling supported by at least one supervisor. Lastly, personal support, is ideally fulfilled by the supervisory team, but can also be (further) provided by peers.

The results of this study can be useful for graduate schools, PhD supervisors, PhD candidates or those considering a PhD. However, this study also comes with limitations. A first limitation of this study is that we only focussed on the experiences of PhD candidates. Recognizing that our findings also have implications for PhD supervisors, future research could enrich these insights by delving into the perceptions and experiences of PhD supervisors. A second limitation is the focus on motivation of PhD candidates who, in the end, were sufficiently motivated to get to the final phase of their PhD. It is noteworthy that this deliberate focus on PhD candidates who almost completed their PhDs was chosen to provide a comprehensive understanding of individuals who successfully navigated the doctoral process. However, perhaps, PhD candidates who dropped out during their PhD might have encountered other challenges and barriers and/or utilized different strategies. Inclusion of dropped-out PhD candidates in future studies can further strengthen insight into the complex nature of motivational development and offer a more nuanced understanding of the diverse motivational dynamics and by that, contribute to a sustainable doctoral environment.

## Conclusion

This study revealed factors that contribute positively and/or negatively to autonomous and controlled motivation during a PhD trajectory and result in the following practical implications to foster AM and reduce CM: (1) PhD candidates and their supervisors should explicitly discuss learning goals and expectations of the PhD trajectory to contribute to a safe learning climate; (2) PhD candidates value to have at least one supervisor who is approachable, makes time, provides constructive and timely feedback, gives trust and autonomy, and sees the person behind the studies; (3) To strengthen peer support, it is important to facilitate peer activities in both formal (e.g. intervision, conferences) and non-formal (e.g. drinks) settings; (4) Autonomy is important during a PhD trajectory and it is necessary to find the right balance in guidance. It is essential to regularly evaluate how much autonomy is needed and it is important to align the amount of guidance accordingly, as the need for autonomy often changes as the PhD candidates gains more experience and expertise; (5) When difficulties are overcome, this is experienced as a personal achievement and success experience. It is important as research team to openly discuss the ‘bumps during the ride’ and stimulate solution seeking approaches. Some factors could coincide and change from positive to negative and vice versa, showing that a successful PhD journey cannot simply be reduced to an absence of negative experiences.

### Supplementary Information

**Additional file 1. **Interview guide (translated to English).

**Additional file 2. **Overview of all emerged themes and sub-themes.

## Data Availability

All data generated or analysed during this study are included in this published article [and its supplementary information files].

## Footnotes

1 den Bakker C, de Beaufort A, Ommering B, Dekker F, Bustraan J. Inspecting the leaky clinician-scientist pipeline – A national study on medical PhD candidates’ motivations in the Netherlands. Manuscript submitted for publication. 2023.

2 den Bakker C, Wijnen-Meijer M, Bustraan J, Dekker F, de Beaufort A. Comparing medical PhD training programmes around the world: a matter of apples and oranges? Manuscript submitted for publication. 2023.
