# Supplementary material for: The bumpy ride to a medical PhD degree: a qualitative study on factors influencing motivation
Source: BMC Med Educ. 2024 Feb 19;24:159. doi: 10.1186/s12909-023-04973-z (PMC10875841; doi:10.1186/s12909-023-04973-z)
Supplement: Supplementary file 1 — Additional file 1. Interview guide (translated to English). [file 12909_2023_4973_MOESM1_ESM.docx]

**Additional file 1– Interview Guide (translated to** **English)**

**Introduction interview**

- Introduction interviewer
- Explaining methods (timeline construction)
- Repeat information stated in the pre-sent information letter (e.g. privacy regulation, etc.)
- Informed consent (recording interview and use of anonymised data in research paper)

**Start of the interview**

- **Prior to starting a PhD trajectory**

1. At which medical school did you study? If applicable; What job(s) did you have after graduation and
 before starting your PhD trajectory? When did you decide you wanted to pursue a PhD? And how did you
 find your current PhD program?

2. What were your motives to initiate a PhD several years ago? What expectations did you have
 of your PhD program? What was your view on doing a PhD? What was (less) appealing?

[motivations & values]

3. At that time, did you already have some ideas about what career you aspired after your PhD? If so, what
 ideas did you have regarding your career after your PhD? Did you (already) have a preferred specialty in
 mind? Did you (already) have ideas about applying for future job positions? (How) Did science play a part in
 your career ambitions? What role for science did you see in your future career? [ambitions]

- **Construction of timeline during the PhD trajectory**

1. Explaining the timeline and different colours of post-its for different phases.
2. Interviewee is writing remarkable, important and meaningful events or experiences on the post-its.
3. Hereafter, the interviewee puts the post-its on the timeline in chronological order. The higher a post-it is pasted on the Y-axis, the more positive the participant looked back on it.

- **Discussing and reflecting on the PhD trajectory timeline**

1. **Start of the PhD trajectory**
   1. You just started your PhD trajectory. Can you tell me something about this time? What were your first impressions? Was it what you expected it to be?
      *[potential in-depth questions regarding work/life factors, support (team), autonomy, research topic and content, culture]*
   2. Can you tell me something about what you wrote on these post-its? How do you think this experience or event might have impacted your motivation for your PhD program and your (academic) ambitions after your PhD?
   3. Overall, how do you look back on this initial phase now?
      *[when only a few post-its have been pasted, potential deepening questions can be: what did you like, like less/dislike, or find difficult? Did your motivation, job satisfaction and/or ambition changed in this initial phase, if so, how come? What were your ambitions for the future at that moment? Looking back at that time, do you wanted things to be different? Is there any advice you would like have to have given yourself at that time?]*
2. **Mid-stage of the PhD trajectory**
   1. We are now continuing to the mid-phase of your PhD trajectory. Can you tell me something about this time?
      *[potential in-depth questions regarding work/life factors, support (team), autonomy, research topic and content, culture]*
   2. Can you tell me something about what you wrote on these post-its? How do you think this experience or event might have impacted your motivation for your PhD program and your (academic) ambitions after your PhD?
      *[potential in-depth questions can be: Were there times when you had serious doubts about whether you were being able to or wanting to finish your PhD? When was it? Where did these doubts came from? If not spontaneously addressed: How would you describe the relationship with your supervisors? How was the collaboration? What role did they fulfill in your trajectory?]*
   3. Overall, how do you look back on this mid-phase of your PhD trajectory now?
      *[when only a few post-its have been pasted, potential deepening questions can be: what did you like, like less/dislike, or find difficult? Did your motivation, job satisfaction and/or ambition changed in this mid-phase, if so, how come? What were your ambitions for the future at that moment? Looking back at that time, do you wanted things to be different? Is there any advice you would like have to have given yourself at that time?]*
3. **Final stage of the PhD trajectory**
   1. We are now progressing to the final and current phase of your PhD trajectory. Can you tell me something about this time? *[potential in-depth questions regarding work/life factors, support (team), autonomy, research topic and content, culture]*
   2. Can you tell me something about what you wrote on these post-its? How do you think this experience or event might have impacted your motivation for your PhD program and your (academic) ambitions after your PhD?
      *[when only a few post-its have been pasted, potential deepening questions can be: Can you tell me something more about how you are doing in this phase? What are your expectations? Do you have any doubts about finishing your PhD trajectory?* *What in particular motivates you in this phase of your PhD trajectory? Are there things you find difficult at this time? What are your ambitions for the future (after your PhD)? (How) Does science play a part in your career ambitions? What role for science do you see in your future career?]*
4. **Finalizing the timeline of the PhD trajectory**
   1. Are there things you would like to add or have not discussed yet?
   2. Can you summarize in a few sentences how your motivation and ambition has changed throughout your PhD trajectory? Can you also indicate what has been decisive for your motivation?
   3. If you could give your younger self any advice at the time you started to consider pursuing a PhD, what advice would you have given yourself?

**End of the interview**

- Reflecting on the interview
- Repeat information about confidentiality, member check etc.
- Check contact information for future contact
- Thank you!

**Check for interviewer:**

1. Clarity (Do I get it? Only one interpretation possible?)

2. Relevance (Does this answer the question?)

3. Completeness

4. Validity (interviewee's opinion)
