# Supplementary material for: The bumpy ride to a medical PhD degree: a qualitative study on factors influencing motivation
Source: BMC Med Educ. 2024 Feb 19;24:159. doi: 10.1186/s12909-023-04973-z (PMC10875841; doi:10.1186/s12909-023-04973-z)
Supplement: Supplementary file 2 — Additional file 2. Overview of all emerged themes and sub-themes. [file 12909_2023_4973_MOESM2_ESM.docx]

**Additional file 2 – Overview of all emerged themes and sub-themes**

| **Themes** | **Sub-themes** |
| --- | --- |
| 1. Initial motivation to start  a PhD matters | 1.1 As stepping stone towards a clinician-scientist career  1.1.1 Longing for extra (academic) challenges next to   clinical tasks (e.g. due to the need ‘to think out of the   box’ instead of following protocols)  1.1.2 Develop research skills   1.1.3 Would like to become an expert on and/or   contribute to the research topic  1.1.4 Research is important for being a good doctor  1.1.5 Research might be more appealing than clinical work   (only)  1.1.6 To get into and get to know the academic world  1.2 As stop-over for career orientation purposes  1.2.1 To buy time for future career steps e.g. specialty   decision  1.2.2 Preferring a PhD over working as DNIT for years  1.3 As vehicle to get into future clinical job positions  1.3.1 Pursuing a PhD to get into the desired (sub)specialty   1.3.2 Pursuing a PhD to increase chances to get into  specialty training 1.4 Others  1.4.1 It (i.e. PhD) came across my path  1.4.2 A PhD degree can only benefit and won’t harm you |
| 2. Autonomy, a matter of the right dose at the right time | 2.1 Autonomy in research projects and initiatives  2.2 (Un)clarity in tasks and expectations  2.3 Need for more guidance 2.4 Pressure to publish (soon) 2.5 (Dis)Liking imposed (clinical) tasks 2.6 Autonomy in (daily) time management within work 2.7 Work life balance |
| 3. PhD as proof of competence and/or as learning trajectory? | 3.1 (Not) Feeling competent (enough) 3.2 Comparing to others  3.3 Urge to stand out and show your competence  3.4 Wants to do well in the eyes of others  3.5 Opportunity for education  3.6 Protected time to (further) develop skills and knowledge |
| 4. It takes (at least) two to tango | 4.1 (Lack of) Commitment and guidance from research team 4.2 (Lack of) Academic guidance  4.3 (Lack of) Mental and personal support 4.4 Supervisor (does not) make(s) time for me 4.5 (Lack of) Clear and constructive feedback 4.6 Credibility supervisor 4.7 (No) Click with supervisor(s) 4.8 Feeling alone in my projects 4.9 Conflicts of interest 4.10 Dependency relationship(s) 4.11 Role model  4.12 Same or different expectations 4.13 Trust in supervisor 4.14 Supervisor is open to my ideas  4.15 Team is proud of my work 4.16 Compassion of team when facing difficulties 4.17 Supervision matching needs 4.18 Feeling safe to talk about PhD struggles with team |
| 5. Peers can make or break your PhD. | 5.1 (Lack of) Relatedness with peers 5.2 (Lack of) Shared experiences with peers  5.3 Peers became friends 5.4 Support from peers 5.5 Informal meetings and activities 5.6 Competitive environment |
| 6. Strategies to stay or get back on track. | 6.1 Active solution-seeking approach  6.1.1 Switch to other supportive working environment  6.1.2 Turning conflicts into positive learning experiences  6.1.3 Transform own experiences into the ambition to do   things differently in future academic career  6.2 Accept that lows are part of a PhD journey  6.2.1 Having the end in view  6.2.2 Last mile is the longest  6.2.3 Finish what you have started  6.2.4 Invested so much time, energy, and effort  6.2.5 Take it as it comes  6.2.6 Not feeling able to change difficulties  6.2.7 Accepting although it was essentially not OK  6.2.8 Not feeling safe to speak up |
|  |  |
